# Antiplasmodial activity of solvent fractions of methanolic root extract of *Dodonaea angustifolia* in *Plasmodium berghei* infected mice

**DOI:** 10.1186/1472-6882-14-462

**Published:** 2014-12-03

**Authors:** Wote Amelo, Pushpa Nagpal, Eyassu Makonnen

**Affiliations:** School of Pharmacy, Jimma University, Jimma, Ethiopia; Department of Pharmacology, College of Health Sciences, Addis Ababa University, Addis Ababa, Ethiopia

**Keywords:** Malaria, Drug resistance, *Dodonaea angustifolia*, *Plasmodium berghei* and LD_50_

## Abstract

**Background:**

Malaria is one of the most important infectious diseases in the World. The choice for the treatment is highly limited, and several of these may eventually be lost or compromised due to drug resistance. The use of plant medicine in the treatment of malaria and its various presentations is a common practice in many countries of Africa where the disease is mostly endemic. *Dodonaea angustifolia* is traditionally used in Ethiopia for prophylaxis against malaria. The present study is attempted to evaluate the antimalarial activity of the solvent fractions of root extracts of *D. angustifolia* in *P. berghei* infected mice*.*

**Methods:**

In this study, 4-days Peter’s suppressive test was used to determine parasite inhibition. Acute toxicity test was also conducted on the most active fraction according to Organization for Economic Cooperation and Development (OECD) guidelines 425. Data was analyzed by using Windows SPSS version 16 and expressed as mean ± SD for each dose level. ANOVA followed by Post Hoc Tukey’s HSD was used to compare result between treatment and control groups. Students paired t-test was employed to test significance for the difference between initial and final results within the same group.

**Results:**

All three fractions showed varying degrees of antiplasmodial activity. The n-butanol fraction displayed a relatively highest suppression of parasitaemia (67.51%) at an oral dose of 600 mg/kg. Lower doses, 200 mg/kg and 400 mg/kg, of the fraction also resulted in parasitaemia suppression of 38.02% and 55.85%, respectively. Chemosuppressive activity of chloroform and aqueous fractions was less compared to that of n-butanol fraction. All the three fractions displayed dose dependent significant (P < 0.001) antiplasmodial activity as compared to the control. Survival time was prolonged in case of n-butanol and chloroform fractions. No lethality to mice was seen with n-butanol fraction up to a dose of 2000 mg/kg.

**Conclusion:**

All the three fractions possessed significant antiplasmodial activity as compared with the control group. n-butanol fraction was found to be the most active fraction with minimal toxicity and might contain potential lead molecule for the development of a new drug for treatment of malaria.

## Background

Malaria, a tropical blood-borne protozoan disease*,* is one of the most important infectious diseases in the World [1]. An estimated 3.3 billion of the total world population live in areas with malaria risk [2] and an estimated death of 660 000. Africa is the most affected continent: accounting for about 90% of all malaria deaths [3].

Malaria control has relied on two arms namely control of the *anopheles* mosquito vector and effective case management [4]. For decades, drug resistance has been one of the main obstacles in the fight against malaria [5]. It is responsible for the spread of malaria to new areas, the recurrence of malaria in areas where the disease had been eradicated and plays an important role in the occurrence and severity of epidemics in some parts of the World [1]. The choice of therapies for the treatment is highly limited, and several of these may eventually be lost or compromised due to drug resistance [6]. Furthermore, the difficulty of creating efficient vaccines and also adverse side-effects of the existing anti-malarial drugs highlight the urgent need for novel, well-tolerated anti-malarial drugs for both prophylaxis and treatment of malaria [7].

According to several reports, up to 80% of world’s populations rely on traditional medicine mainly on herbal remedies as primary source of medicinal agents for the treatment of diseases [8]. The use of phytomedicine in the treatment of malaria and its various presentations is a common practice in many countries of Africa where the disease is mostly endemic [9]. Some antimalarial drugs in use today (quinine and artemisinin) were either obtained from plants or developed using their chemical structures as templates [10]. This explains why a lot of current research focuses on natural molecules and plant-derived products as they can be sourced easily, locally available and can be selected on the basis of their ethnomedicinal use [11]. In Ethiopia it is estimated that about 80% of the Ethiopian population is still dependent on traditional medicine, which essentially involves the use of plants [12].

*Dodonaea angustifolia* (Sapindaceae) commonly referred to as Sand Olive, kitkita (Amharic), Hitacha (Afan Oromoo) and itancha (Sidamu afoo). It is a shrub or small tree with narrow shiny pale green leaves with a distinctive small winged fruit [13]. It grows at the altitudes between 800 and 2650 m above sea level and in areas with a rainfall range of 500-1500 mm/year, commonly up to a height of 8 m. It grows on dry rocky slopes between 1500 and 2100 m throughout Ethiopia [13]. It also grows in a variety of habitats and rapidly colonizes open areas of recently cleared forests [14]. The genus *Dodonaea* comprises about 60 species [15]. The centre of origin of *D. angustifolia* is believed to be Australia, but it is also widely distributed throughout the tropics and subtropics [16].

The plant was selected on the basis of its use in folk medicine for malaria treatment/prophylaxis and its promising antimalarial activity from previous *in vitro* and *in vivo* studies. This plant has a wide range of therapeutic applications like pneumonia and other pulmonary diseases including tuberculosis [16], analgesic and antipyretic effect [17]. In Ethiopia the plant is used for wound dressing, and for treatment of skin diseases, fever, sore throat, rhinitis, sinusitis, and influenza [18] and also for prophylaxis against malaria, bacteria and helminthes [19]. Different parts of *D. angustifolia* has been reported to have significant antimalarial activity*,* methanolic extract of root 84.52% with 600 mg/kg dose [12], aqueous extract of seeds, 79.45% with 1000 mg/kg dose [19], methanolic extract of leaves, 80.89% with 300 mg/kg dose [20].

*D. angustifolia* contains several secondary metabolites like quinines, saponins, flavonoides, alkaloids, terpenoids, diterpenoids, phenols and essential oils [12, 16]. From the studies conducted on the plant until now, it is known that the methanolic root extract of the plant has schizonticidal activity as high as 84.52% [12]. Thus, the present study aims at evaluating the *invivo* antiplasmodial activity of solvent fractions of methanolic root extract of *Dodonaea angustifolia*.

## Methods

### Collection of plant materials

The roots of *Dodonaea angustifolia* (Sapindaceae) was collected from Chuko, Ethiopia. After botanical identification, a voucher specimen, WA01/2011, was deposited at the national herbarium, College of Natural Sciences, Addis Ababa University.

### Crude extract preparation and fractionation of the crude extract

The collected plant materials were washed with distilled water and air-dried at room temperature.

The plant material was then ground to powder using an electrical grinding mill and kept till extracted with solvent. The crude extract was prepared by cold maceration technique, by refluxing 800 g of plant material in methanol (98%). After 72 hrs the mixture was filtered using Whatman filter paper. The extract was concentrated in a rota vapor and stored in a refrigerator until fractionation.

The crude extract was in distilled water and Tween 80 (3%). Then the suspension was shaken in a separatory funnel by adding n-butanol each time 3 times and the n-butanol fraction was obtained. The aqueous residue was then shaken with chloroform 3 times to obtain the chloroform fraction. The chloroform fraction was then treated similarly as n- butanol fraction. The n-butanol and chloroform fractions were concentrated in rota vapor. The aqueous residue was also lyophilized to obtain the aqueous fraction. Then the fractionations were kept in an amber glass bottle and stored in a refrigerator.

### Animal housing

Adult male Swiss albino mice (25- 32 g), aged 6- 8 weeks were obtained from Ethiopian Health and Nutrition Research Institute (EHNRI). The animals were kept in cages and housed in a standard animal house under natural 12/12 h light dark cycle at room temperature. They were maintained on standard pelleted diet and water *ad libitum*. Before the experiment was started, they were all acclimatized to the test environment. The study protocol for animal experiment was submitted to and approved by the Ethics committee of the Department of Pharmacology, School of Medicine. The animals were then randomly assigned to the control and experimental groups.

### *In vivo*anti-malarial sensitivity against *P. berghei*

Tests were performed in a 4-day suppressive standard test [21]. Donor *Plasmodium berghei* infected mice were killed by head blows and the blood was collected by heart puncture. Then the blood was diluted with normal saline so that each 0.2 ml contained approximately10^7^ parasite infected erythrocytes [21]. The mice were divided in to five groups of five in each for each fraction. Three groups of mice received the fractions, while the other two groups were used as standard and control groups.

On day 0 (before starting administration of the test substance and standard), mice of all groups were inoculated intraperitoneally, with 0.2 ml of infected blood containing about 1 × 10^7^ parasitized red blood cells, which is expected to produce steadily rising consistent infection of the required intensity in mice [21]. The mice in the test groups received fractions of the extract once daily for 4 days. The control group was administered with distilled water and the standard group with chloroquine phosphate (10 mg/kg) [21]. For each group of the animals 0.2 ml of the preparation was administered and gavage was used for oral administration.

On day 4 after infection, a thin smear of blood film was taken from the peripheral blood of the tail of each mouse in the test and control groups. The smears were fixed with methanol and then stained with Giemsa stain. Then, each stained slide was microscopically examined under oil immersion of 1000 magnification (1000×) power to evaluate the mean % of parasitaemia and suppression of each fraction in comparison with control group. The mean parasitaemia was calculated and expressed as follows [21]:


Percentage parasitaemia suppression was calculated according to the following formula [21]


### Determination of mean survival time

Mortality was monitored daily and the number of days from the time of inoculation of the parasite up to death was recorded for each mouse in all groups throughout the follow up period. The mean survival time (MST) for each group was calculated as follows [16]:


### Determination of packed cell volume

The packed cell volume (PCV) of each mouse was measured before infection and on day 4 after infection [22]. For this purpose, blood was collected from tail of each mouse in heparinized microhaematocrit capillary tubes up to 3/4th of their length. The tubes were sealed by critoseal and placed in a microhaematocrit centrifuge (Gelman Hawksley, England) at 5000 rpm for 5 minutes. Then the tubes were taken out from the centrifuge and the result was read using microhaematocrit reader (Gelman Hawksley, England) according to the following formula [16]:


### Acute toxicity test

A pilot study was conducted to identify the most active fraction and acute toxicity of the fraction was tested. Healthy female swiss albino mice maintained under standard laboratory conditions were used for acute toxicity test according to Organization for Economic Cooperation and Development (OECD) guidelines. The acute toxicity studies were conducted as per the OECD guidelines 425 [23] where the limit test dose of 2000 mg/kg was used. They were all observed for toxicity signs like changes in physical appearance, behavioral change, motor and feeding activities, and other signs of acute toxicity and mortality after administration of the dose.

### Data analysis

To analyze data obtained during the experiment windows SPSS version 16 was used and expressed as mean ± SD for each dose level. ANOVA followed by Post Hoc Tukey’s HSD was used to compare result between treatment and control groups. Students paired t-test was employed to test significance for the difference between initial and final results within the same group. The result was considered statistically significant at 95% confidence level and P-value <0.05.

## Results

### Acute toxicity

The n-butanol fraction was tolerated by the study mice when administered orally. No death was observed in the animals receiving the fraction up to a dose of 2000 mg/kg body weight. This shows that the lethal dose (LD_50_) is greater than 2000 mg/kg body weight.

### Effect of fractions of methanolic root extract of *Dodonaea angustifolia*on parasitemia and mean survival time

The results of the 4-day suppressive test of the n-butanol fractions at different dose levels on parasitaemia and survival time in mice infected with *Plasmodium berghei* are summarized in Table 1. The mice treated with CQ were completely free from the parasites on day four. All mice treated with the n-butanol fraction of methanolic root extract of *D. angustifolia* significantly reduced parasitaemia level as compared to the control group (P < 0.001) and the difference was also significant in comparison with standard drug treated group. The fraction produced varying degrees of chemosuppressive effect in a dose dependent manner. The highest suppression (67.51%) was seen with the dose of 600 mg/kg body weight. However the mice were not completely cured from the infection in all treatment doses but did significantly prolonged the mean survival time at all dose levels (P < 0.05)

**Table 1:** **Effect of n-butanol fraction of methanolic root extract of** ***D. angustifolia*** **on percent parasitaemia and mean survival time of the mice**

Treatment	Dose mg/kg	%Parasitaemia	%Chemosuppression	Mean survival time
n-butanol fraction	200	34.02 ± 2.29	38.02^c^	10.33 ± 1.76^a^
	400	24.24 ± 3.42	55.85^c^	10.79 ± 1.37^a^
	600	17.83 ± 4.11	67.51^c^	11.37 ± 1.61^b^
Chloroquine	10	0	100	14.19 ± 0.84
Distilled water	1 ml	54.90 ± 4.39	0	7.79 ± 0.73

a = P < 0.05, b = P < 0.01, c = P < 0.001, parasitaemia and survival time expressed as mean ± SD, n = 5, the results are expressed as the percent suppression of parasitaemia with reference to non-treated mice. distilled water = control.

Significant reduction of parasitaemia (P < 0.001) was observed in all groups of mice treated with chloroform fractions of methanol root extract of *D. angustifolia* compared with the control group (Table 2). The percent chemosuppression with the doses of 200, 400 and 600 mg/kg body weight of the mice was 26.1, 36.6 and 42.00, respectively (Table 3). The highest suppression was seen with the dose of 600 mg/kg body weight of the mice.

**Table 2:** **Effect of chloroform fraction of the methanolic root extract of** ***D. angustifolia*** **on parasitaemia and mean survival time of the mice**

Treatment	Dose mg/kg	%Parasitemia	%Chemosuppression	Mean survival time
Chloroform fract.	200	40.58 ± 1.91	26.10^c^	8.96 ± 1.05
	400	34.79 ± 0.50	36.64^c^	9.99 ± 0.71^a^
	600	31.85 ± 4.62	42.00^c^	10.75 ± 2.16^b^
Chloroquine	10	0	100	12.40 ± 0.54
Distilled water	1 ml	56.55 ± 8.38	0	7.63 ± 0.85

a = P < 0.05, b = P < 0.01, c = P < 0.001, parasitaemia and survival time expressed as mean ± SD, n = 5, the results are expressed as the percent suppression of parasitaemia with reference to non-treated mice, distilled water = control.

**Table 3:** **Effect of aqueous fraction of the methanolic root extract of** ***D. angustifolia*** **on parasitaemia and mean survival time of the mice**

Treatment	Dose mg/kg	%Parasitemia	%Chemosuppression	Mean survival time
Aq. fraction	200	48.79 ± 2.00	14.76^c^	7.40 ± 0.51
	400	47.56 ± 1.56	16.91^c^	8.29 ± 0.83
	600	45.23 ± 1.31	20.98^c^	8.58 ± 0.53
Chloroquine	10	0	100	12.84 ± 1.32
Distilled water	1 ml	57.24 ± 2.85	0	7.58 ± 0.53

c = p < 0.001, parasitaemia and survival time expressed as mean ± SD, n = 5, The results are expressed as the percent suppression of parasitaemia with reference to non-treated mice.

The same table shows that the fraction also prolonged the mean survival time of the study mice significantly (P < 0.05) at 400 and 600 mg/kg body weight as compared with the mice in control group.

The aqueous fraction suppressed parasitaemia significantly at all dose levels (P < 0.001) as compared with the control group (Table 3). The mean survival time, however, did not significantly change with the aqueous fraction at any of the dose levels.

### Effect of fractions of methanolic root extract of *D. angustifolia*on body weight

As shown in Table 4, a significant (p < 0.05) loss of body weight was observed between days 0 and 4 in all fraction treated groups. All the mice which received the three fractions showed statistically significant difference (P < 0.05) in body weights at day 4 as compared with their weight at day 0. Butanol fraction displayed significant difference in all doses as compared with control group, but the difference was not significant in comparison with standard drug treated group. Chloroform and aqueous fractions showed no difference in comparison with both control and standard drug treated group.

**Table 4:** **Effect of the fractions of methanolic root extract of** ***Dodonaea angustifolia*** **on the body weight of the mice**

Test substance	Dose (mg/kg)	Body weight (kg)		%change
		D-0	D-4	
Butanol	200	30.42 ± 0.68	29.18 ± 0.21	-4.25^c^
	400	29.84 ± 0.99	28.66 ± 0.44	-4.11^c^
	600	28.70 ± 1.06	27.60 ± 0.34	-3.98^a^
Chloroquine	10	29.42 ± 2.17	29.04 ± 1.63	-1.30
Distilled water	1 ml	28.72 ± 0.77	25.74 ± 0.45	-11.58
Chloroform	200	29.74 ± 1.46	27.80 ± 1.92	-6.98
	400	29.50 ± 1.75	27.06 ± 0.83	-9.02
	600	29.56 ± 1.81	27.20 ± 1.10	-8.68
Chloroquine	10	29.38 ± 1.04	29.36 ± 1.32	-0.07
Distilled water	1 ml	30.34 ± 0.92	26.54 ± 1.39	-14.32
Aq. fraction	200	30.26 ± 1.30	28.18 ± 0.64	-7.38
	400	30.98 ± 0.93	28.34 ± 0.42	-9.31
	600	29.40 ± 2.32	27.36 ± 0.46	-7.46
Chloroquine	10	28.84 ± 1.39	28.46 ± 2.24	-1.34
Distilled water	1 ml	31.46 ± 0.83	27.06 ± 0.79	-16.26

a = P < 0.05, c = P < .001, Body weight expressed as mean ± SD, n = 5 the results are expressed as the percent reduction of body weight with reference to day 0. Distilled water = control, D0 = a day infection initiated, D4 = 5^th^ day of infection.

### Effect of fractions of the methanolic root extract of *D. angustifolia*on PCV

**Figure 1:**
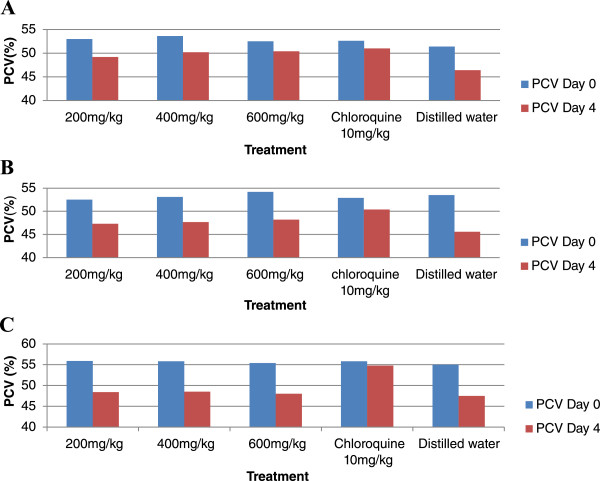
**Effect of fractions (A = butanol, B = chloroform and C = aqueous fraction) of the methanolic root extract of** ***D. angustifolia*** **on PCV of** ***P. berghei*** **infected mice**.

The effect of the fractions of the extract on PCV on days 0 and 4 is indicated in Figure 1. The aqueous fraction showed significant difference (P < 0.01) in PCV as compared with the standard drug treated group but no difference in comparison with untreated group. The difference displayed by n-butanol and chloroform fractions was not significant in comparison with standard drug (chloroquine) treated group. Comparison of PCV at day 0 and day 4 indicated that all the three fractions did not prevent PCV reduction (P < 0.05).

## Discussion

Plants contain chemical constituents that have great potential for medicinal use and both traditional healers and pharmaceutical drug companies make use of these plants [24]. The extracts of *D. angustifolia* were reported to contain different classes of secondary metabolites such as: tannins, alkaloids, quinines, saponins, flavonoides, terpenoids, diterpenoids, phenols and essential oils [12, 16]. Terpenoids, flavonoids saponins and alkaloids were known to have antiplasmodial activity [1, 25, 26]. The phenol present in this plant which has antioxidant effect [27] may also contribute to the antiplasmodial activity. Antioxidative activity can inhibit haem polymerization as haem has to be oxidized before polymerization, and the unpolymerized haem is very toxic for the intraerythrocytic plasmodia [6]. These chemical compounds which are also found in the fractions may be acting singly or in synergy with one another to exert the observed antiplasmodial activity of the fractions.

The n-butanol fraction did not show mortality within 24 hours up to a dose of 2000 mg/kg body weight indicating that the fraction is safe. In general, if the lethal dose (LD_50_) of the test substance is three times more than the minimum effective dose, the substance is considered a good candidate for further studies [28]. This result is in agreement with other studies done on the same plant with crude extracts, indicating that no death was observed with different dose levels, 1000 mg/kg body weight [19], 3000 mg/kg body weight [12] and 4500 mg/kg body weight [16]. But abnormalities like depression, weakness and rough hair coat was observed with 4500 mg/kg crude methanolic extract of *D. angustifolia* [12].

The fractions from this study showed varying degrees of antiplasmodial activity. The n-butanol fraction showed a relatively highest suppression of parasitaemia (67.51%) at an oral dose of 600 mg/kg (Tab 1). Administration of 200 mg/kg and 400 mg/kg doses to mice with also resulted in parasitaemia suppression. All three test doses displayed significant difference (P < 0.001) in comparison with control group and the standard drug (chloroquine) treated group. The activity may be due to the individual or synergistic effect of the secondary metabolite found in the fraction like alkaloids, saponins, flavonoids, tannins and phenols [29–31]. The chloroform fraction also showed significant (P < 0.001) antiplasmodial activity (Tab 2). The fraction showed dose dependent chemosuppression. The aqueous fraction similarly displayed a dose-dependent suppressive activity (Tab 3). But the chemosupressive activity of chloroform and aqueous fraction was, however, less as compared with that of n-butanol fraction indicating the difference in the type and concentration of the secondary metabolites in the fractions. The same result was reported from the study done on different plant but the same solvents for fractionation [32]. This may be due to less number of secondary metabolites that are active against malaria parasites in aqueous fraction.

In this study the chemosuppressive activity of all three fractions is lower than the activity obtained from other studies of the same plant with the reported results of crude methanolic extracts of leaves (80.89%) [20], crude aqueous extracts of seed (79.45%) [19] and crude methanolic extract of roots (84.52%) [12]. This finding is in agreement with another study in which the crude extract of *Cocholospermum tinctorum* reported to show better *in vivo* antimalarial activity than the fractions [33]. The reduction in activity of the crude extracts upon fractionations could be explained by the loss of synergistic action among the chemical compounds in an extract, and by denaturation of the compounds during storage.

The n-butanol and chloroform fractions prolonged the mean survival time of the study mice indicating that they suppressed *P. berghei* and reduced the overall pathologic effect of the parasite on the study mice. But all doses from aqueous fraction were not able to significantly prolong the mean survival time as compared with control group. This may be due to the less schizoticidal activity of the fraction as compared to the other two fractions.

Animals suffer from anemia because of red cell destruction, either by parasite multiplication or by spleen reticuloendotelial cell action [34]. In this study the n-butanol, chloroform and aqueous fractions did not show protection against reduction in PCV as compared with day 0 (Figure 1). In untreated mice, the parasite count increased and the PCV decreased markedly which was also observed in previous studies [26, 35].

Body weight loss is one feature of rodent malaria infections [16]. All the three fractions didn’t prevent weight reduction in the present study (Tab 4). This result is in agreement with one of the previous studies on the crude extracts of the same plant [16]. However, the result of the present study on body weight is not in agreement with the findings from the crude extracts of *Asparagus africanus* [35] and butanol fraction of both crude aqueous and hydroalcoholic extract of *C. myricoides* [26] which prevented body weight loss of *P. berghei* infected mice. The inconsistency of the results might be due to variation in nutrient content of the plants. The loss of body weight of the mice treated with the fractions may possibly be due to the appetite suppressive effect of the components in the fraction.

## Conclusion

From the present study it could be concluded that all the three fractions possessed significant antiplasmodial activity as seen in their ability to suppress *P. berghei* infection in mice. Butanol fraction was found to be relatively the most active fraction with minimal toxicity. So, the n-butanol fraction might contain potential lead molecule for the development of a new drug for treatment of malaria. The present study confirmed the finding of previous studies conducted on the same plant.
